# Cost-Effectiveness of Aprepitant in Preventing Chemotherapy-Induced Nausea and Vomiting: A Systematic Review of Published Articles

**DOI:** 10.3389/fpubh.2021.660514

**Published:** 2021-08-25

**Authors:** Tingting Qiu, Peng Men, Tong Sun, Suodi Zhai

**Affiliations:** ^1^Department of Pharmacy, Peking University Third Hospital, Beijing, China; ^2^Institute for Drug Evaluation, Peking University Health Science Center, Beijing, China; ^3^Department of Pharmacy, Aviation General Hospital, Beijing, China

**Keywords:** aprepitant, nausea, vomiting, cost, effectiveness

## Abstract

**Objectives:** The aim of this systematic review is to assess the published cost-effectiveness analyses of aprepitant for patients with chemotherapy-induced nausea and vomiting (CINV).

**Methods:** A systematic literature search was performed on PubMed, EMbase, the Cochrane Library, CNKI, WANFANG DATA, and CBM database. The date of publication is up to January 2019. Two reviewers independently reviewed titles, abstracts, and articles sequentially to select studies for data abstraction based on the inclusion and exclusion criteria. Disagreements were resolved and reviewers reached a consensus. The quality of the included studies was assessed according to the 24-item checklist of the consolidated health economic evaluation reporting standards (CHEERS). The costs reported by the included studies were converted to US dollars *via* purchasing power parities (PPP) in the year 2019 using the CCEMG–EPPI–Certer Cost Converter.

**Results:** Thirteen articles were included based on the inclusion criteria for cost-effectiveness analysis and cost-utility analysis. Twelve studies were rated as good quality and one as a moderate quality based on the CHEERS checklist. Eight studies compared aprepitant plus 5-hydroxytryptamine-3 receptor antagonist (5-HT3RA) and dexamethasone with the standard regimen (5-HT3RA and dexamethasone). It was concluded that aprepitant plus standard regimen was a cost-effective strategy for preventing CINV. Only one study that compared aprepitant plus 5-HT3RA with 5-HT3RA, concluded that the addition of aprepitant reduced the incidence of severe nausea, and it might also provide an economic benefit in the overall management. Four studies that compared aprepitant with other antiemetic drugs concluded that aprepitant is a cost-effective strategy for preventing CINV compared with metoclopramide. However, netupitan + palonosetron and olanzapine are cost-effective compared with aprepitant.

**Conclusion:** This study is the first systematic evaluation of adding aprepitant to standard regimens for patients with CINV. Most economic evaluations of antiemetic medications are reported to be of good quality. Adding aprepitant to standard regimens is found to be a cost-effective strategy for preventing CINV.

## Introduction

Chemotherapy-induced nausea and vomiting (CINV) is a common side effect of chemotherapy. The prevalence of CINV has been estimated to be as high as 70–80% without appropriate antiemetic prophylaxis (1). Patients who receive highly emetogenic chemotherapy (HEC) and moderately emetogenic chemotherapy (MEC) are the major populations who suffer from nausea and vomiting (2). CINV can be classified as the following: acute (occurs within the first 24 h after chemotherapy initiation) and delayed (occurs within 24–120 h postchemotherapy) events.

Aprepitant, a neurokinin-1 receptor antagonist (NK-1RA), has been showing effectiveness in preventing CINV. Adding to standard antiemetic regimens (a 5-hydroxytryptamine-3 receptor antagonist (5-HT3RA) and/or a glucocorticoid), aprepitant has been proved to lead to a further decrease in the incidence of CINV than the standard regimen alone (3–6).

Currently, the guidelines of the National Comprehensive Cancer Network (NCCN) (7), the American Society of Clinical Oncology (ASCO) (8), and the Multinational Association of Supportive Care in Cancer (MASCC)/European Society of Medical Oncology (ESMO) (9) endorsed the use of NK-1RAs plus as a standard regimen in patients who received HEC for preventing CINV. However, the ASCO and MASCC/ESMO guidelines did not recommend NK-1RA for MEC patients. In contrast, the NCCN guideline recommended that an NK-1RA should be added to a standard regimen for patients with additional risk factors or previous treatment failure with a standard regimen alone. As for the Chinese guideline (10), NK-1RA was recommended for MEC patients based on particular situations.

Chemotherapy-induced nausea and vomiting can significantly affect the adherence of patients to cancer treatments and impair the quality of life (11). Uncontrolled CINV can also increase health care expenditure and resource utilization (12). Although optimal antiemetic prophylaxis, according to the emetogenic risk of chemotherapy, is important for patients to continue their cancer treatment, the increased financial burden is a concern for aprepitant, which is a costly antiemetic agent. Gomez et al. (11) reported that socioeconomic barriers associated with NK1RA therapy affected suboptimal adherence to guideline recommendations for antiemetic prophylaxis. While many studies have reported on the cost-effectiveness of aprepitant for treating CINV, a systematic review of economic evaluations of aprepitant is currently lacking. Therefore, it is necessary to conduct a comprehensive systematic evaluation and analysis of the existing economic research evidence of aprepitant, assess the cost-effectiveness of adding aprepitant to standard regimens for patients with CINV, and provide support for clinical rational drug use and medical insurance decision-making.

## Methods

A systematic review was conducted following the preferred reporting items for systematic reviews and metaanalyses (PRISMA) guidelines (12). It was registered on the International Prospective Register for systematic reviews (No. CRD 42020152060).

### Search Methods for Identification of Studies

A literature search was performed using the following databases: PubMed, Embase, the Cochrane Library, and three Chinese databases (China National Knowledge Infrastructure [CNKI], WANFANG DATA, and Chinese Biomedical Literature Database [CBM]). The search time was from the date of establishment of the databases to January 2019. The following search terms were used, “aprepitant,” “emend,” “cost,” “effectiveness,” “utility,” “benefit,” “economic,” “expenses,” and “pharmacoeconomic.”

### Criteria for Considering Studies

#### Types of Participants

Patients diagnosed with malignant tumors by histopathology and/or cytology who received HEC or MEC.

#### Types of Interventions

Aprepitant plus 5HT3RA with or without dexamethasone for the prevention of CINV.

#### Types of Comparators

The following comparisons were acceptable for evaluation:

**·**Aprepitant regimen (aprepitant, 5-HT3RA and dexamethasone) vs. standard regimen (5-HT3RA and dexamethasone);

**·**Aprepitant plus 5-HT3RA vs. 5-HT3RA;

**·**Aprepitant vs. other antiemetic drugs.

#### Types of Outcomes

We evaluated the incremental cost-effectiveness ratio (ICER) measure as the primary outcome, and incremental effectiveness and incremental cost measures as the secondary outcome.

#### Types of Studies

Pharmacoeconomic studies were included if: (1) full texts were published in any language; (2) economic evaluations (including cost-effectiveness, cost-utility, cost-minimization, cost-benefit analyses, and cost analysis). Exclusion criteria were as follows: review articles, editorials and opinions, letters, research protocols, conference abstracts, notes and books.

### Selection of Included Studies

Articles retrieved from the literature search were independently screened based on the title and abstract by two authors (TTQ and PM). Studies that did not meet the criteria were excluded. After the initial screening, two researchers (TTQ, PM) independently assessed the full texts of eligible citations. The list of included studies was reached by a consensus. Any disagreements were resolved by discussion or by consulting with a senior author (SDZ).

### Data Extraction

Data extraction was performed using predesigned data extraction tables in Microsoft Excel. For all studies, the following information: authors, published year, country, type of model, perspective, model details (time horizon, discount rate), source of funding, sensitivity or uncertainty analysis, incremental effectiveness and costs, and ICER were extracted.

### Reporting Quality Assessment

The quality of the pharmacoeconomic studies was assessed by a 24-item checklist of the Consolidated Health Economic Evaluation Reporting Standards (CHEERS) statement, which was used as a checklist to rate the quality of reporting in the included papers. The CHEERS statement of the International Society for Pharmacoeconomics and Outcomes Research (ISPOR) Health Economic Evaluation Publication Guidelines Good Reporting Practices Task Force is a guideline intended to improve reporting of the economic evaluation (13, 14). The quality of the included studies was evaluated by the answers to the questions, which were “yes” (reported and scored 1) or “no” (not reported and scored 0) or “partly” (partially reported and scored 0.5) or “NA”(not applicable and scored 1). The studies were separated into four quality categories. Those studies that fulfilled 100% of the items were classified as excellent quality; those that fulfilled between 75 and 100% of the items were classified as good quality; those that fulfilled between 50 and 75% of the items were classified as moderate quality; and those that fulfilled ≤50% of the items were classified as low quality (15, 16).

### Strategy for Data Synthesis

To facilitate the comparison of ICERs, all costs were converted into US dollars *via* purchasing power parities (PPP) in the year 2019. PPP was defined as the rates of currency conversion that eliminate the differences in price levels between countries. PPP conversion factors were obtained from the Organization for Economic Co-Operation and Development Stat database (17). We converted the original cost estimates to the target currency and price year.

## Results

### Selection of Studies

**Figure 1:**
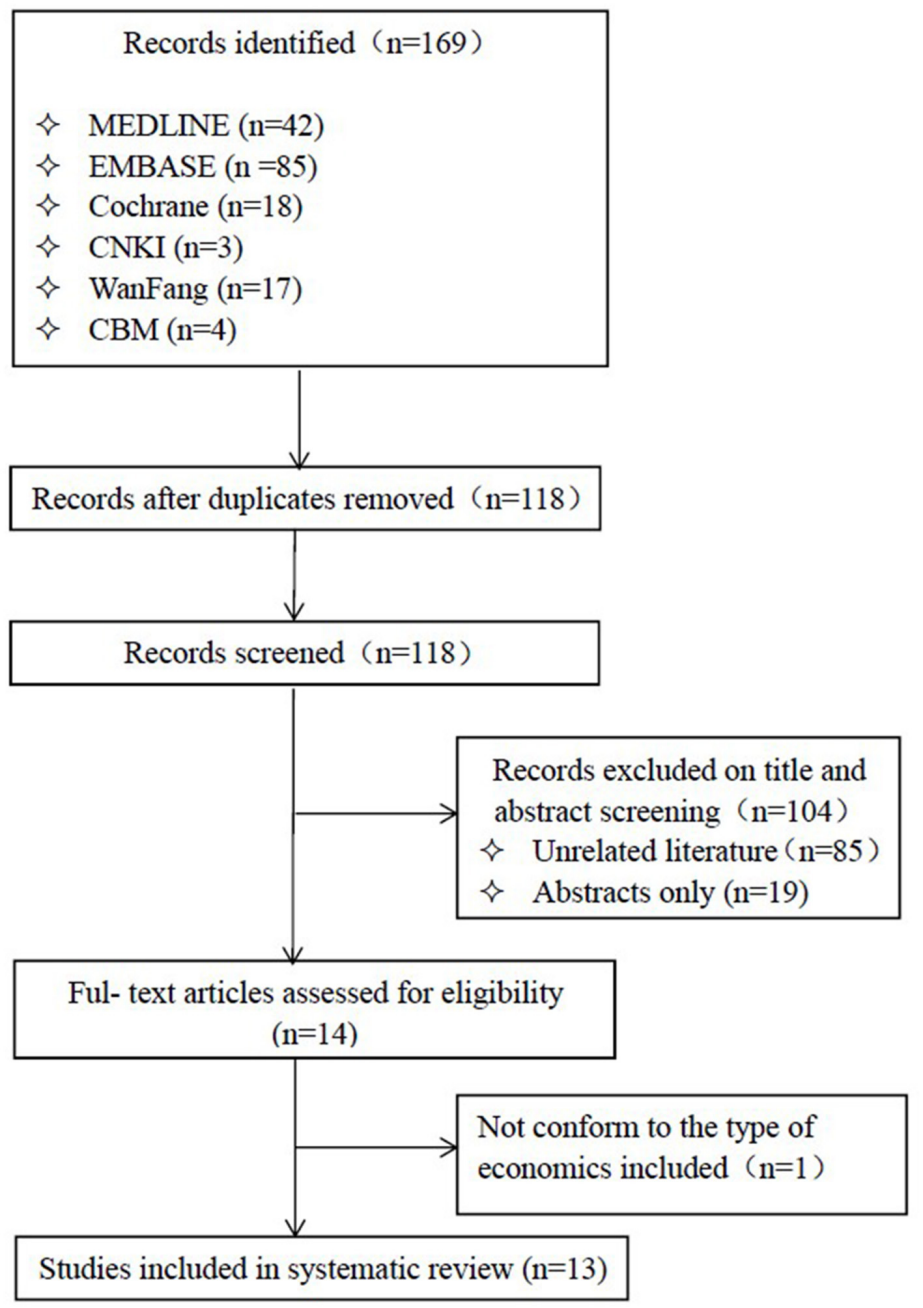
Study selection process.

After a thorough search of the databases, we acquired 169 articles, of which 104 were excluded based on the title and abstract screening. A total of 13 published studies (18–30) were selected for final inclusion (Figure 1) after reviewing.

### General Characteristics of the Included Studies

Table 1 describes the general characteristics of the included studies. Of the 13 studies, eight (18, 21–26, 30) conducted both cost-effectiveness analysis (CEA) and cost-utility analysis (CUA), two studies conducted CUA (19, 20), one study conducted CEA (27), one study conducted cost-consequences analysis (CCA) (28), and one study conducted both CUA and budget impact analysis (29).

**Table 1:** General characteristics of the included studies.

**References**	**Country**	**Economic type**	**Model type**	**perspective**	**Time horizon**	**Participants using APR**	**Comparison**	**Sources of effectiveness and safety data**
Annemans et al. (18)	Belgium	CEA CUA	Decision analytical model	Belgium health-care system	21 days/cycle, 4 cycles	cisplatin-based chemotherapy regimens and MEC regimens	ARP+OND+DEX PLA+OND+DEX	Two RCT (5, 31)
Avritscher et al. (19)	USA	CUA	Markov model	USA third-party payer	21 days/cycle, 4 cycles	AC regimens	OND+DEX OND+DEX+APR after emesis PAL+DEX PAL+DEX+ APR after emesis OND+DEX+ARP PAL+DEX+ARP	Five RCT (6, 31–34)
Cawston et al. (20)	UK	CUA	Markov model	UK NHS payer	5 days	Patients receiving HEC and MEC.	NEPA ARP+PAL PAL	Systematic review and meta-analysis (37RCT) by the authors themselves
Chan et al. (21)	Hong Kong	CEA CUA	Decision analytical model	Hong Kong Public Healthcare System.	5 days	Cisplatin-based chemotherapy anthracycline and AC chemotherapy	ARP+OND+DEX OND +DEX GRA+DEX TRO+DEX	Four RCT (3–5, 35)
Chanthawong et al. (22)	Southeast Asia	CEA CUA	Decision analytical model	Societal perspective method	5 days	HEC in outpatient setting, platinum-based and AC -based regimen	DEX + 5HT3RA DEX + 5HT3RA +OLN DEX + 5HT3RA +APR	Systematic review and network meta-analysis (24RCT) by the authors themselves
Humphreys et al. (23)	UK	CEA CUA	Decision analytical model	UK National Health Service (NHS)	5 days	Patients receiving MEC	ARP+OND+DEX MET+OND+DEX	RCT (35)
Lopes et al. (24)	Singapore	CEA CUA	Decision analytical model	Singapore health care system	5 days	Cisplatin regimen, AC regimen	ARP+5HT3RA+DEX OND (GRA)+DEX	Four RCT (3–5, 35)
Lordick et al. (25)	Germany	CEA CUA	Decision analytical model	Patient's and statutory health insurance	5 days	HEC in outpatient cisplatin regimen	ARP+OND+DEX PLA+OND+DEX	A combined analysis of two multicentre, randomized, double-blind phase III clinical trials (36)
Moore et al. (26)	USA	CEA CUA	Markov model	Payer	28 days/cycle, 5 cycles	Cisplatin-based HEC	ARP+OND+DEX OND+DEX+ aprepitant after CINV	RCT (3)
Nakamura et al. (27)	Japan	CEA	——	——	——	CINV during high-dose chemotherapy (HDCT)	APR+GRA GRA	Retrospectively reviewed medical records of National Hospital Organization Nagoya Medical Center
Ravasio et al. (28)	Italy	CCA	Decision analytical model	Italian National Health Service payer	5 days	Cisplatin-based HEC	ARP+OND+DEX OND+DEX	RCT (5)
Restelli et al. (29)	Italy	CUA	Markov model	Italian National Health Service (NHS)	5 days/cycle, at least six cycles	Patients receiving HEC and MEC.	NEPA (for HEC and MEC) APR + PAL (for HEC and MEC) APR + OND (for HEC) fAPR + PAL (for HEC and MEC) fAPR + OND (for HEC) PAL (for HEC and MEC)	Three RCT (37–39)
Tsukiyama et al. (30)	Japan	CEA CUA	Decision analytical model	Japanese National Health Insurance system payer	5 days	Cisplatin-containing HEC	APR+GRA+DEX GRA+DEX	RCT (40)

*AC, anthracycline and cyclophosphamide; HEC, highly emetogenic chemotherapy; MEC, moderately emetogenic chemotherapy; CINV, chemotherapy-induced nausea and vomiting; ARP, aprepitant; DEX, dexamethasone; OND, ondansetron; PAL, palonosetron; GRA: granisetron; TRO, tropisetron; OLN, olanzapine; fAPR, fosaprepitant; NEPA, netupitant + palonosetron*.

Ten of the 13 economic studies included data of clinical outcomes from randomized controlled trials. The clinical data of Chanthawong et al. (22) and Cawston et al. (20) came from systematic review and metaanalysis. Nakamura et al. (27) acquired data from a retrospective analysis of direct medical costs of National Hospital Organization Nagoya Medical Center between January 2009 and December 2013.

Of the 13 studies included, eight of used the decision analytical model (18, 21–25, 28, 30), four used the Markov model (19, 20, 26, 29), and one study did not use model analysis (27).

Eleven studies were conducted from a payer perspective, of which eight were performed from a public payer perspective (e.g., National Health Service, National Health Insurance system, and health-care system) (18, 20, 21, 23, 24, 28–30). In contrast, one study used a patient and statutory health insurance perspective (25), and two studies used the perspective of the payer, but did not describe it specifically (19, 26). One study used a societal perspective (22) and one study (27) did not mention it.

The time horizon for two studies (18, 19) was four cycles (21 days in each cycle), for another eight studies (20–25, 28, 30) time horizon was 5 days, for one study (26) it was five cycles (28 days in each cycle), time horizon of one study (29) was at least six cycles (5 days in each cycle) when CUA was conducted and 5 years when budget impacted analyses, and one study (27) did not mention the time horizon.

Among the included studies, the most common comparison was the aprepitant triple regimen (aprepitant+5-HT3RA+glucocorticoid) vs. standard regimen (5-HT3RA+glucocorticoid) with or without placebo (18, 19, 21, 24–26, 28, 30), the other comparison was aprepitant +5HT3RA vs. aprepitant (27), and the comparison of positive comparators such as netupitan + palonosetron (PAL) (NEPA) (20, 29), metoclopramide (23), and olanzapine (22). Most studies reported ICERs as cost-effectiveness evaluation outcomes.

Three of the studies were funded by Merck & Co (21, 23, 25), one was funded by Sanofi-aventis (19), one was funded by Helsinn Healthcare SA (20), one was funded by MSD Italia srl (28), one was funded by Italfarmaco Spa (29), and one was supported by a Health Outcomes Research Starter Grant from the PhRMA Foundation (26). Two studies (18, 24) did not disclose the funding source, although some authors of these studies were employees from pharmaceutical companies, and some authors received funding or honoraria from pharmaceutical companies. The remaining three studies (22, 27, 30) did not mention any funding information at all.

### Quality of the Included Studies

Twelve studies were of good quality based on the CHEERS checklist (18–26, 28–30), one was of moderate quality (27). The results of the quality assessment of the included studies are shown in Table 2.

**Table 2:** Quality of the economic evaluations (as assessed by the CHEERS statement).

**Item no**.	**Section/item**	**Annemans et al. (18)**	**Avritscher et al. (19)**	**Cawston et al. (20)**	**Chanet al. (21)**	**Chanthawong et al. (22)**	**Humphreys et al. (23)**	**Lopes et al. (24)**	**Lordick et al. (25)**	**Moore et al. (26)**	**Nakamura et al. (27)**	**Ravasio et al. (28)**	**Restelli et al. (29)**	**Tsukiyama et al. (30)**
1	Title	Yes	Yes	Yes	Yes	Yes	Yes	Yes	Yes	Yes	Yes	Yes	Yes	Yes
2	Abstract	Yes	Partly	Yes	Yes	Yes	Yes	Yes	Yes	Yes	Yes	Yes	Yes	Partly
3	Background and objectives	Yes	Yes	Yes	Yes	Yes	Yes	Yes	Yes	Yes	Yes	Yes	Yes	Yes
4	Target population and subgroups	Yes	Yes	Yes	Yes	Yes	Yes	Yes	Yes	Yes	Yes	Yes	Yes	Yes
5	Setting and location	Yes	Yes	Yes	Yes	Yes	Yes	Yes	Yes	Yes	Yes	Yes	Yes	Yes
6	Study perspective	Yes	Yes	Yes	Yes	Yes	Yes	Yes	Yes	Yes	No	Yes	Yes	Yes
7	Comparators	Yes	Yes	Yes	Yes	Yes	Yes	Yes	Yes	Yes	Yes	Yes	Yes	Yes
8	Time horizon	Yes	Yes	Yes	Yes	Yes	Yes	Yes	Yes	Yes	No	Yes	Yes	Yes
9	Discount rate	NA	NA	NA	NA	NA	NA	NA	NA	NA	NA	NA	NA	NA
10	Choice of health outcomes	Yes	Yes	Yes	Yes	Yes	Yes	Yes	Yes	Yes	Yes	Yes	Yes	Yes
11	Measurement of effectiveness	Yes	Partly	Yes	Yes	Yes	Yes	Yes	Yes	No	Yes	Yes	Yes	Yes
12	Measurement and valuation of preference-based outcomes	Yes	Yes	Yes	Yes	Yes	Yes	Yes	Yes	Yes	Yes	Yes	Yes	Yes
13	Estimating resources and costs	Yes	Yes	Yes	Yes	Yes	Yes	Yes	Yes	Yes	Yes	Yes	Yes	Yes
14	Currency, price date, and conversion	Partly	Yes	Yes	Yes	Yes	Partly	Partly	Yes	Yes	Yes	Yes	Yes	Yes
15	Choice of model	Yes	Partly	Partly	Partly	Partly	Partly	Partly	Partly	Partly	NA	Partly	Partly	Partly
16	Assumptions	Yes	Yes	Yes	No	Yes	Yes	No	No	Yes	NA	Yes	Yes	Yes
17	Analytic methods	Yes	Yes	Yes	Yes	Yes	Yes	Yes	Yes	Yes	Yes	Yes	Yes	Yes
18	Study parameters	Yes	Yes	Yes	Yes	Yes	Yes	Yes	Yes	Yes	NA	Yes	Yes	Yes
19	Incremental costs and outcomes	Yes	Yes	Yes	Yes	Yes	Yes	Yes	Yes	Yes	No	No	Yes	Yes
20	Characterizing uncertainty	Yes	Yes	Yes	No	Yes	Yes	No	No	Yes	No	Yes	No	Yes
21	Characterizing heterogeneity	No	No	No	No	No	No	No	No	No	No	No	No	No
22	Study findings, limitations, generalizability, and current knowledge	Partly	Yes	Yes	Yes	Yes	Yes	Yes	Yes	Yes	Yes	Yes	Yes	Yes
23	Source of funding	No	Yes	Yes	Yes	No	Yes	No	Yes	Yes	No	Yes	Yes	No
24	Conflicts of interest	No	Yes	Yes	Yes	Yes	Yes	Yes	Yes	No	Yes	Yes	Yes	Yes
Overall quality		Good	Good	Good	Good	Good	Good	Good	Good	Good	Moderate	Good	Good	Good

*CHEERS, Consolidated Health Economic Evaluation Reporting Standards*.

*Yes, reported and scored 1*.

*No, not reported and scored 0*.

*Partly, Partlyially reported and scored 0.5*.

*NA, not applicable and scored 1*.

We found that the included studies did not report several items on the CHEERS checklist. One of the studies (27) did not mention the perspective of the research, and five studies (21, 24, 25, 27, 29) did not state the results of uncertainty analyses, both the items on the CHEERS checklist that should be reported in the abstracts of economic evaluations.

Two studies used the perspective of the payer but did not describe it specifically (19, 26), and one study (27) did not mention the perspective nor did it use the model analysis.

Reasons for the choice of the economic model were not reported in most of the studies. Two studies did not perform a sensitivity analysis (21, 27). Even some studies that achieved good quality ratings did not meet the checklist criterion for characterizing heterogeneity. The roles of the funders play in the identification, design, conduct, and reporting of the analysis were not reported in all the included studies.

We also synthesized the results of quality rating and the funding information (Table 3). However, no relationship between sources of funding and the quality of the included studies was identified in this review.

**Table 3:** Funding information and overall quality of the economic evaluations.

**References**	**Research fund from pharmaceutical companies^a^**	**Research fund from other sources**	**Authors act as employees from pharmaceutical companies**	**Authors receive funds or honoraria from pharmaceutical companies^b^**	**Overall quality**
Annemans et al. (18)	Not mentioned	Not mentioned	Merck Sharp & Dohme^c^	Not mentioned	Good
Avritscher et al. (19)	Sanofi-aventis	Not mentioned	Not mentioned	MGI Pharma, Inc., GlaxoSmithKline, Sanofifi-aventis, and Merck^c^	Good
Cawston et al. (20)	Helsinn Healthcare SA	Not mentioned	Helsinn Healthcare SA^c^	Helsinn Healthcare SA	Good
Chan et al. (21)	Merck & Co	Not mentioned	Not mentioned	Not mentioned	Good
Chanthawong et al. (22)	Not mentioned	Not mentioned	Not mentioned	Not mentioned	Good
Humphreys et al. (23)	Merck and Co, Dohme Corp, a subsidiary of Merck and Co	Not mentioned	Merck Sharp and Dohme Ltd and Merck and Co^c^^,^^d^	Merck Sharp and Dohme Ltd, Roche, Novartis, Celgene Corp, GlaxoSmithKline, Amgen, and Pfizer Inc^c^	Good
Lopes et al. (24)	Not mentioned	Not mentioned	Merck & Co, Merck Shap & Dohme^c^	Merck Sharp & Dohme^e^, GSK^e^, MSD^d^	Good
Lordick et al. (25)	Merck & Co	Not mentioned	MSD Deutschland GmbH^c^, Merck research laboratories^c^	MSD Sharp & Dohme GmbH^c^^,^^d^	Good
Moore et al. (26)	Not mentioned	A Health Outcomes Research Starter Grant from the PhRMA Foundation	Not mentioned	Not mentioned	Good
Nakamura et al. (27)	Not mentioned	Not mentioned	Not mentioned	Not mentioned	Moderate
Ravasio et al. (28)	MSD Italia srl.	Not mentioned	Not mentioned	Not mentioned	Good
Restelli et al. (29)	Italfarmaco Spa	Not mentioned	Italfarmaco Spa^c^	Bayer and Italfarmaco^d^, Italfarmaco Spa, Zambon Spa, Helsinn Healthcare SA and Polichem SA^c^	Good
Tsukiyama et al. (30)	Not mentioned	Not mentioned	Not mentioned	Not mentioned	Good

a *Authors declared that the research was funded by the pharmaceutical company*.

b *Authors who were not employees of pharmaceutical companies who received funding or honoraria from the companies*.

c *Co-author*.

d *First author*.

e *Corresponding author*.

### Economic Evaluation and Sensitivity Analysis Results

The economic outcomes of the included studies are summarized in Tables 4–**6**.

**Table 4:** Summary of economic evaluation outcomes comparing aprepitant regimen (aprepitant, 5-HT3RA and dexamethasone) vs. standard regimen (5-HT3RA and dexamethasone).

**References**	**Comparison**	**Incremental effectiveness:**	**Incremental costs**	**Incremental costs (2019 US)**	**Original ICER (per QALY)**	**Threshold of ICER (per QALY)**	**Threshold of ICER (2019 US per QALY)**	**ICER (2019 US $ per QALY)**	**Sensitivity or uncertainty analysis**
Annemans et al. (18)	PLA+OND+DEX ARP+OND+DEX	CR: 0.11 (HEC trial-based) 0.11 (HEC real-life-based) 0.13 (MEC trial-based) 0.13 (MEC real-life-based) QALY: 0.003 (HEC trial-based) 0.003 (HEC real-life-based) 0.014 (MEC trial-based) 0.014 (MEC real-life-based)	–€66.84 (HEC trial based) –€74.62 (HEC real-life based) –€17.95 (MEC trial based) –€21.70 (MEC real-life based)	–$101.67 (HEC trial based) –$113.51 (HEC real-life based) –$27.30 (MEC trial based) –$33.01 (MEC real-life based)	Dominant Dominant Dominant Dominant	NA	NA	NA	One-way sensitivity analyses: Robust: cost of emesis, the clinical benefit of Aprepitant. Sensitive: decrease in cost of ondansetron for the MEC.
Avritscher et al. (19)	OND+DEX OND+DEX+APR After emesis PAL+DEX PAL+DEX+APR After emesis OND+DEX+ARP PAL+DEX+ARP	—— 0.0021 0.0051 0.0016 0.0006 0.0044	—— $366 $589 $319 $159 $603	—— $436.11 $701.82 $380.10 $189.46 $718.50	—— $174,286 $115,490 $199,375 Dominated $200,526	$50,000 –$100,000	$59577.35 –$119154.69	—— $207,669.94 $137,611.75 $237,564.66 NA $238,936.13	One-way sensitivity analysis: Sensitive: values of antiemetic effectiveness and of the probability of emesis-related hospitalization. Probabilistic sensitivity analysis: using the $100,000/QALY benchmark, the palonosetronbased two-drug strategy and the two-drug regimen plus aprepitant following emesis were shown to be cost-effective in 39% and 26% of the Monte Carlo simulations.
Chan et al. (21)	OND +DEX GRA+DEX TRO+DEX ARP+OND+DEX	1. Cisplatin-based HEC analysis with 5HT3RA administered on Day 1 only: 0.001716 2. Cisplatin-based HEC analysis with 5HT3RA administered Day 1–4: 0.000942 3. AC-based HEC analysis with 5HT3RA administered Day 1–3: 0.00122	1. OND: HK$411.33 TRO: HK$466.69 GRA: HK$463.48 2. OND: HK$415.27 TRO: HK$338.01 GRA: HK$95.31 3. OND: HK$235.11 TRO: HK$107.67 GRA: HK$ −55.20	1. OND: $83.28 TRO: $94.49 GRA: $93.84 2. OND: $84.08 TRO: $68.44 GRA: $19.30 3. OND: $47.60 TRO: $21.80 GRA: –$11.18	1. OND: HK$239,644 TRO: HK$271,901 GRA: HK$270,031 2. OND: HK$440,950 TRO: HK$358,910 GRA: HK$101,202 3. OND: HK$195,442 TRO: HK$89,506 GRA: Dominated	HK$798,078	$161584.90	1. OND: $48,520.13 TRO: $55,051.13 GRA: $54,672.52 2. OND: $89,278.07 TRO: $72,667.63 GRA: $20,490.12 3. OND: $39,570.66 TRO: $18,122.06 GRA: NA	NA
Lopes et al. (24)	OND (GRA)+DEX ARP+5HT3RA+DEX	1. Cisplatin regimen with single-day 5-HT3 RA as comparator: 0.00017 2. Cisplatin regimen with 4-d 5-HT3 RA as comparator: 0.00094 3. AC regimen with 3-d 5-HT3 RA as comparator: 0.0012	1. OND: SGD58 GRA: SGD68 2. OND: SGD63 GRA: SGD25 3. OND: SGD91 GRA: Cost-saving	1. OND: $74.52 GRA: $ 87.37 2. OND: $ 80.94 GRA: $32.12 3. OND: $116.92 GRA: Cost-saving	1. OND: SGD48440 GRA: SGD49778 2. OND: SGD58719 GRA: SGD22636 3. OND: SGD21421 GRA: Cost-saving	SGD160,000	$205574.46	1. OND: $62,237.67 GRA: $63,956.78 2. OND: $75,444.54 GRA: $29,083.65 3. OND: $27,522.57 GRA: NA	One-way and two-way sensitivity analyses: relatively insensitive to changes in the cost inputs.
Lordick et al. (25)	PLA+OND+DEX ARP+OND+DEX	0.0017	€49.6	$78.10	€28891	€43600	$68648.12	$45,488.83	One-way sensitivity analyses: most sensitive to costs of hospitalisations and rescue medication, whereas the variation of outpatient costs only slightly impacted on the incremental cost-effectiveness ratios.
Moore et al. (26)	OND+DEX ARP+OND+DEX OND+DEX+ aprepitant if CINV in previous cycle	—— 0.007 0.003	—— $682 $289	—— $876.44 $371.39	—— $97,429 $96,333	$50,000	$64255.16	—— $125,206.31 $123,797.84	Univariate sensitivity analyses: robust Probabilistic sensitivity analysis: 98.86% of the samples were not cost-effective, the ICER for the three-drug strategy was $100,516/QALY (95% confidence range $90,396/QALY –$111,239/QALY).
Ravasio et al. (28)	OND+DEX ARP+OND+DEX	NA	– €1.43	–$2.06	NA	NA	NA	NA	Sensitivity and threshold analyses confirmed the base case results.
Tsukiyama et al. (30)	GRA+DEX APR+GRA+DEX	0.00159	Costs JPY (USD): Outpatient setting 6,192 (56.92) Inpatient setting 9,820 (90.27)	Costs JPY (USD): Outpatient setting 64.02 (60.36) Inpatient setting 101.52 (95.73)	ICER JPY/QALY (USD/QALY): Outpatient setting 3906698 (35910) Inpatient setting 6195781 (56952)	5 million JPY [45960 USD] in Japan and 50,000 USD in the USA	$48741.44 in Japan and $53025.94 in the USA	Outpatient setting $40,389.23 Inpatient setting $64,054.82	Univariate sensitivity analyses: sensitive to cost of the aprepitant regimen, CR rate of the delayed phase, utility weight of CP, and CR rate of the acute phase. Probabilistic sensitivity analysis: aprepitant regimen was cost-effective was higher in the OCS than in the ICS. A dot in the first quadrant means that the aprepitant regimen is both costlier and more effective than the nonaprepitant regimen.

*AC, anthracycline and cyclophosphamide; HEC, highly emetogenic chemotherapy; MEC, moderately emetogenic chemotherapy; DEX, Decamethasone; OND, Ondansetron; GRA, Granisetron; TRO, Tropisetron; ICER, incremental cost-effectiveness ratio; QALY, quality-adjusted life-years; OCS, outpatient care setting; ICS, in the inpatient care setting; CR, complete response; CP, complete protection; Dominant, less cost and more effective; Dominated, less effective and more expensive*.

#### Aprepitant Regimen vs. Standard Regimen

Eight studies compared aprepitant regimen (aprepitant, 5-HT3RA, and dexamethasone) vs. standard regimen (5-HT3RA and dexamethasone), two of which used placebo (18, 25). The drugs of 5-HT3RA are ondansetron (OND), granisetron (GRA), PAL, and tropisetron (TRO). Six studies used the Decision analytical model (18, 21, 24, 25, 28, 30), and two used the Markov model (19, 26).

Eight studies concluded that aprepitant regimen was cost-effective compared with standard regimen. Among these eight studies, three were conducted in Europe [Belgium (18), Germany (25), and Italy (28)], two were from North America (USA (19, 26)), and three were from Asia [Hong Kong (21), Singapore (24), and Japan (30)].

Table 4 summarized the comparisons of case-based ICER values adjusted to year 2019 US$ values, and the results of the sensitivity analyses. Among them, sensitivity analysis was not performed in one study (21). One of the eight studies (28) did not analyze quality-adjusted life years (QALYs), but it conducted cost-consequence analysis, and the remaining seven studies all analyzed QALYs. Two studies (18, 19) showed that the ICER of aprepitant ranged from US$207,669.94 per QALY to US$ 238,936.13(adjusted to the year 2019 value) for a 21-day time horizon. One study (26) showed that the ICER of aprepitant was US$125,206.31 per QALY (adjusted to year 2019 value) for a 28-day time horizon. The other studies (21, 24, 25, 30) indicated that the ICER of aprepitant ranged from US$ 18,122.06 per QALY to US$ 89,278.07 per QALY (adjusted to year 2019 value) for a 5-day time horizon.

Two studies, Chan et al. (21) and Lopes et al. (24), were developed under the following three scenarios:

Scenario 1. Patients receiving cisplatin-based chemotherapy who received the aprepitant-containing regimen were compared with a standard regimen in which the 5HT3RA was administered on day 1 only;

Scenario 2. Patients receiving cisplatin-based chemotherapy who received the aprepitant-containing regimen were compared with a standard regimen in which the 5HT3RA was administered on days 1–4;

Scenario 3. Patients receiving an AC-based chemotherapy who received the aprepitant-containing regimen were compared with a standard regimen in which the 5HT3RA was administered on days 1–3.

These two studies (21, 24) came to the same conclusion that aprepitant-containing regimen was associated with higher acquisition costs but lower costs relating to patient emesis-related management, hospitalization, and use of rescue medication.

The results of eight studies suggested that adding aprepitant to standard regimen (5HT3RA+dexamethasone) was a cost-effective strategy for preventing CINV.

#### Aprepitant Plus 5-HT3RA vs. 5-HT3RA

Only one study, Nakamura et al. (27), compared aprepitant plus 5-HT3RA with a 5-HT3RA (GRA) (Table 5). This study did not mention the perspective of the research and time horizon. It did not use model analysis and did not perform a sensitivity analysis. The study data was from a retrospective analysis of direct medical costs. It also conducted cost-effectiveness analysis. The research was of moderate quality according to the 24 questions of the CHEERS checklist. The guidelines recommend the treatment of CINV by comparing the aprepitant triple regimen with the standard regimen. Aprepitant plus 5-HT3RA vs. 5-HT3RA was not common. In the studies of Nakamura et al. (27), the total mean cost per patient during hospitalization was USD 19,052.33 (adjusted to the year 2019 value) in the aprepitant group and USD 22306.81(adjusted to the year 2019 value)in the non-aprepitant group. Although this difference was not statistically significant (*p* = 0.077), it indicated that the use of aprepitant reduced the total medical expense by USD 3,254.48 (adjusted to the year 2019 value) per patient. This lower cost in the aprepitant group was due to the shorter hospitalization period and reduced costs for transfusion and infection treatment. This study indicated that the addition of aprepitant for CINV prophylaxis during high-dose chemotherapy (HDCT) reduced the incidence of severe nausea and might also provide an economic benefit in the overall management of HDCT.

**Table 5:** Summary of economic evaluation outcomes comparing aprepitant plus 5-HT3RA Vs. 5-HT3RA.

**Reference**	**Comparison**	**Incremental effectiveness:**	**Incremental costs**	**Original ICER (per QALY)**	**Incremental costs (2019 US)**	**Threshold of ICER (per QALY)**	**Threshold of ICER (2019 US per QALY)**	**ICER(2019 US $ per QALY)**	**Sensitivity or uncertainty analysis**
Nakamura et al. (27)	GRA APR+GRA	The incidence of severe nausea (≥grade 3) was significantly lower in the aprepitant group than in the non-aprepitant group (*p* = 0.039).	–$2947.7	NA	–$3254.48	NA	NA	NA	NA

*ICER, incremental cost-effectiveness ratio; QALY, quality-adjusted life-years; GRA, Granisetron; APR, aprepitant*.

#### Aprepitant vs. Other Antiemetic Drugs

Table 6 summarized the economic results and the sensitivity analysis results of the comparison of aprepitant with other antiemetic drugs.

**Table 6:** Summary of economic evaluation outcomes comparing aprepitant versus other antiemetic drugs.

**References**	**Comparison**	**Incremental effectiveness**	**Incremental costs**	**Incremental costs (2019 US)**	**Original ICER (per QALY)**	**Threshold of ICER (per QALY)**	**Threshold of ICER (2019 US per QALY)**	**ICER (2019 US $ per QALY)**	**Sensitivity or uncertainty analysis**
Cawston et al. (20)	ARP+PAL NEPA	NEPA: 0.001	NEPA: –£44.40	NEPA: -$70.33	Dominant	£30,000	$47523.27	NA	One-way sensitivity analysis: robust Probabilistic sensitivity analysis: in HEC patients, NEPA was a dominant strategy in 89.2% of simulations against APPA and was cost saving but less effective in 10.4% of cases.
Restelli et al. (29)	HEC: APR+PAL NEPA APR+OND NEPA MEC: APR+PAL NEPA	HEC: APR+PAL —— NEPA +0.261 APR+OND —— NEPA +0.077 MEC: APR+PAL —— NEPA +0.052	HEC: APR+PAL —— NEPA—€30.2 APR+OND —— NEPA—€48.4 MEC: APR+PAL—— NEPA—€27.2	HEC: APR+PAL —— NEPA –$42.71 APR+OND —— NEPA –$68.23 MEC: APR+PAL —— NEPA—$38.34	Dominant	€40 000	$56385.71	NA	One-way sensitivity analyses: robust
Chanthawong et al. (22)	DEX + 5HT3RA +APR DEX + 5HT3RA +OLN	OLN: 0.0005	OLN: USD 60.91	OLN: $64.60	Cost-saving	SGD 73,000– USD 50,474	$89628.11 –$53528.63	NA	One-way sensitivity analysis: sensitive in Singapore. Probabilistic sensitivity analysis:The probability of being cost-effective at a cost-effectiveness threshold of 1 GDP/capita varies from 14.7 to 85.2% across countries.
Humphreys et al. (23)	MET+OND+DEX ARP+OND+DEX	APR: 0.0048	APR: £10.32	APR: $16.91	£10,847	£20,000–£30,000	$32777.79 –$49166.69	$17,777.04	Probabilistic sensitivity analysis: probability of the aprepitant regimen being cost-effective, compared with the UK comparator regimen, is 79 and 92% at “willingness-to-pay” thresholds of £20,000/QALY and £30,000/QALY, respectively

*ICER, incremental cost-effectiveness ratio; QALY, quality-adjusted life-years; Dominant, less cost and more effective; NEPA, Netupitant(NETU, 300 mg)+Palonosetron(PA, 0.5 mg); OLN, Olanzapine; PAL, Palonosetron; MET, Metoclopramide; DEX, Decamethasone; OND, Ondansetron*.

##### Aprepitant vs. NEPA

Netupitan + palonosetron is a fixed-dose combination of netupitant (NETU, 300 mg), a new NK-1RA with a long half-life period of 90 h, and PAL (0.5 mg) (41).

Cawston et al. (20) showed that in HEC patients, the NEPA strategy was more effective than APPA (QALDs of 4.263 vs. 4.053; incremental emesis-free, and CINV-free days of +0.354 and +0.237, respectively) and costed less ($126.73 vs. $196.43) (adjusted to the year 2019 value). The result showed that NEPA is the dominant strategy. NEPA was cost-effective for MEC patients, cumulating in an estimated 0.182 extra QALDs at an incremental cost of $10.53 (adjusted to the year 2019 value) compared with PA.

Restelli et al. (29) showed that NEPA is more effective and less expensive (dominant) compared with Aprepitant (APR) + PAL (for HEC and MEC) and APR + OND (for HEC). The use of NEPA would lead to a 5-year cost decrease of $89.8 million (60.2 million for HEC and $29.5 million for MEC) (adjusted to the year 2019 value).

The results of two studies (20, 29) suggest that NEPA is cost-effective for preventing CINV associated with HEC and MEC.

##### Aprepitant vs. Olanzapine

Studies have reported the advantages of olanzapine, an atypical antipsychotic drug, in improving the control of acute and delayed CINV. Chanthawong et al. (22) switched aprepitant to olanzapine and yielded additional 0.0005 QALY with a cost saving of USD 64.60 in Singapore (adjusted to the year 2019 value). This study suggests that the use of olanzapine as part of standard antiemetic regimen is cost-effective for the prevention of CINV in patients receiving HEC.

##### Aprepitant vs. Metoclopramide

Humphreys et al. (23) showed that 5 days after chemotherapy, 64% of patients who received the aprepitant regimen [aprepitant + OND + Decamethasone (DEX)] and 47% of those who received the UK comparator regimen (metoclopramide+OND+DEX) had a complete response to antiemetic therapy (no emesis and no rescue antiemetic therapy). A mean of $60.82 (adjusted to the year 2019 value) (78%) of the cost of aprepitant was offset by reduced health care resource utilization costs. The predicted gain in QALYs of the aprepitant regimen was 0.0048. The ICER of aprepitant, relative to the UK comparator, was $17777.04/QALY, which is well below the threshold commonly accepted in the UK($32777.79–$49166.69/QALY) (adjusted to the year 2019 value). The results of this study suggest that aprepitant is cost-effective for preventing CINV associated with chemotherapy for patients with breast cancer in the UK health care setting.

## Discussion

### Quality of the Economic Evaluations

We systematically searched for, assessed, and summarized the available literature on the cost-effectiveness of aprepitant in patients with CINV. The quality assessment of reviewed articles indicated that most articles were of good and moderate quality. Some studies did not report the reasons for choosing the time horizon or discount rate, and no included studies had a subgroup analysis. We found that only one study (22) in this review used a societal perspective; most studies considered a payer perspective. Societal perspective is the gold standard of pharmacoeconomic studies because it incorporates all costs and health outcomes, although other perspectives may be better for some decision-making situations (42, 43).

The role of funders in the identification, design, implementation, and reporting of research is critical to ensure that readers can reliably detect any potential bias. We found that seven of the included studies received funding from pharmaceutical companies. One study (26) was supported by a Health Outcomes Research Starter Grant from the PhRMA Foundation. Two studies (18, 24) did not disclose the funding source, but some authors were employees from pharmaceutical companies and some authors received funding or honoraria from pharmaceutical companies. Three studies (22, 27, 30) did not mention funding information at all. We found no relationship between the funding sources and the quality of the included studies that were identified in this review.

### Evidence for Cost-Effectiveness

Ten of the 13 economic studies were highly consistent. The aprepitant regimen (APR+5-HT3RA+DEX) is more economical than the standard regimen (5-HT3RA+DXE), APR+5-HT3RA is more economical than 5-HT3RA, and APR+5-HT3RA+DEX is cost-effective for preventing CINV when compared with MET+ 5-HT3RA+DEX. Although aprepitant brings higher drug costs, the costs associated with vomiting management (such as patient management, hospitalization, and costs associated with the use of rescue drugs) are lower in the aprepitant group. Therefore, the aprepitant triple regimen is a cost-saving strategy.

The other three studies were Cawston et al. (20), Restelli et al. (29) on NEPA economics research and Chanthawong et al. (22) on olanzapine. NEPA is recommended for HEC by the NCCN, ASCO, and the MASCC/ ESMO, but as the drug is not yet on the market in China, it is not recommended in the 2014 edition of the Guidelines for the Prevention and Treatment of Vomiting Related to Cancer Therapy (7–10).

Chanthawong et al. (22) showed that compared with triplet antiemetic regimen, switching aprepitant to olanzapine increased QALY and saved costs. The use of olanzapine as part of standard antiemetic regimen is cost-effective for the prevention of CINV in patients receiving HEC in multiple SEA countries. The 2019 version of NCCA recommends two options for olanzapine for HEC, one is in combination with PAL and dexamethasone, and the other is in combination with NK1RA, 5HT3RA, and dexamethasone (7). The 2016 version of ESMO/MASCC recommends that olanzapine seems to be useful in the prophylaxis of delayed nausea [superior to (fos)aprepitant] and equal to (fos)aprepitant in the prevention of acute symptoms. Olanzapine may be considered with a 5-HT3 RA plus dexamethasone, particularly when nausea is an issue, but when using the 10-mg dose, patient sedation may be a concern [MASCC level of confidence: low; MASCC level of consensus: low; ESMO level of evidence II; ESMO grade of recommendation: B] (9). The ASCO considers that olanzapine lacks high-quality efficacy and safety studies, and hence is not recommended (8). There is no recommendation for olanzapine in China guidelines (10), and there are no indications for preventing CINV in the olanzapine instructions. Olanzapine should be used with caution in older people (44). The use of olanzapine for CINV did not reach the consensus of national guidelines. Therefore, even if olanzapine showed economic advantages when compared with aprepitant, the advantages and disadvantages should be considered in clinical decision-making. More studies are needed to analyze olanzapine or NEPA compared with aprepitant.

### Strengths and Limitations

This study has several strengths. First, this review is the first comprehensive synthesis of the evidence of cost-effectiveness for aprepitant in preventing CINV. Second, this review includes all published cost-effectiveness studies of aprepitant, and adjusts all cost-related values of different time and countries to 2019 dollars for better comparison.

This study also has several limitations. First, because of heterogeneity in the methodology (e.g., different types of economic models, time horizon, and perspective) and data sources (e.g., effectiveness and safety data, and costing data) of economic evaluations, it is impossible to combine the data (45). Since it is difficult to compare different economic evaluations and reach an overall conclusion regarding the results, reasoning and conducting quantitative analysis (metaanalysis) is impossible. Thus, the explicit and precise estimation of the reported indicators was not possible, so this issue should be considered for using and generalizing the results.

Second, we analyzed the results of economic assessments conducted in different countries with different health care systems and reimbursement mechanisms, and most studies did not use real-world data. Methods such as cost-benefit thresholds, budgeting, and reimbursement should be taken into account, and so the interpretation of the results should be cautious.

## Conclusions

This is the first systematic review of the cost-effectiveness of aprepitant for people with CINV. Based on the available literature, we drew a conclusion that in patients with CINV, aprepitant as an add-on treatment may represent a cost-effective option compared with standard regimen.

## Data Availability Statement

The original contributions presented in the study are included in the article/supplementary material, further inquiries can be directed to the corresponding author/s.

## Author Contributions

TQ and PM are responsible for the main research and writing of the article. TS is responsible for modifying the article. SZ is the manager of this research and also the corresponding writer. All authors contributed to the conception and design of this work, and approved the final manuscript.

## Conflict of Interest

The authors declare that the research was conducted in the absence of any commercial or financial relationships that could be construed as a potential conflict of interest.

## Publisher's Note

All claims expressed in this article are solely those of the authors and do not necessarily represent those of their affiliated organizations, or those of the publisher, the editors and the reviewers. Any product that may be evaluated in this article, or claim that may be made by its manufacturer, is not guaranteed or endorsed by the publisher.
